# The potential role of synovial cells in the progression and treatment of osteoarthritis

**DOI:** 10.1002/EXP.20220132

**Published:** 2023-07-10

**Authors:** Zaijun Zou, Han Li, Kai Yu, Ke Ma, Qiguang Wang, Junnan Tang, Guozhen Liu, Khoon Lim, Gary Hooper, Tim Woodfield, Xiaolin Cui, Weiguo Zhang, Kang Tian

**Affiliations:** ^1^ Department of Sports Medicine The First Affiliated Hospital of Dalian Medical University Dalian Liaoning China; ^2^ Department of Bone and Joint Central Hospital of Zhuang He City Dalian Liaoning China; ^3^ Department of Clinical Medicine China Medical University Shenyang Liaoning China; ^4^ National Engineering Research Center for Biomaterials Sichuan University Chengdu Sichuan China; ^5^ Department of Cardiology The First Affiliated Hospital of Zhengzhou University Zhengzhou Henan China; ^6^ School of Medicine The Chinese University of Hong Kong (Shenzhen) Shenzhen Guangdong China; ^7^ Christchurch Regenerative Medicine and Tissue Engineering Group (CReaTE) Department of Orthopaedic Surgery and Musculoskeletal Medicine University of Otago Christchurch New Zealand; ^8^ Key Laboratory of Molecular Mechanisms for Repair and Remodeling of Orthopaedic Diseases Liaoning Province Dalian Liaoning China

**Keywords:** cartilage, inflammation, osteoarthritis, synovial cells, synovium, treatment

## Abstract

Osteoarthritis (OA), the commonest arthritis, is characterized by the progressive destruction of cartilage, leading to disability. The Current early clinical treatment strategy for OA often centers on anti‐inflammatory or analgesia medication, weight loss, improved muscular function and articular cartilage repair. Although these treatments can relieve symptoms, OA tends to be progressive, and most patients require arthroplasty at the terminal stages of OA. Recent studies have shown a close correlation between joint pain, inflammation, cartilage destruction and synovial cells. Consequently, understanding the potential mechanisms associated with the action of synovial cells in OA could be beneficial for the clinical management of OA. Therefore, this review comprehensively describes the biological functions of synovial cells, the synovium, together with the pathological changes of synovial cells in OA, and the interaction between the cartilage and synovium, which is lacking in the present literature. Additionally, therapeutic approaches based on synovial cells for OA treatment are further discussed from a clinical perspective, highlighting a new direction in the treatment of OA.

## Graphical abstract

Synovial cells play an important role in the progression of osteoarthritis (OA). This article summarizes the biological functions of the synovium, synovial cells, and synovial fluid, discussing their potential roles in OA. Preclinical and clinical studies related to targeting synovial cells and synovial mesenchymal stem cell (SMSC)‐based treatments are also included. This paper will be beneficial for clinicians seeking to further understand the potential role of synovial cells in the progression and treatment of OA.

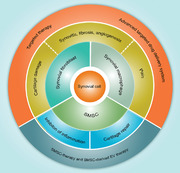

## 1 INTRODUCTION

Osteoarthritis (OA) is a chronic joint disease that affects over 500 million patients around the world, accounting for 7% of the global population.^[^ ^1^ ^]^ In addition, the overall number of patients is expected to rise due to the aging population.^[^ ^2^ ^]^ OA induces joint swelling and limits joint function, severely impacting patients' quality of life and increasing the socioeconomic burden.^[^ ^3^ ^]^ Risk factors, including age, gender, obesity, metabolic diseases, and alterations in lower extremity alignment, together with joint trauma and instability, are etiological factors in the development of OA.^[^ ^4^ ^]^ Generally, the pathological progression of OA involves all of the anatomic components inside and outside the joint, such as the articular cartilage, synovium, synovial fluid, subchondral bone, and periarticular tissues.^[^ ^5^ ^]^

**FIGURE 1:**
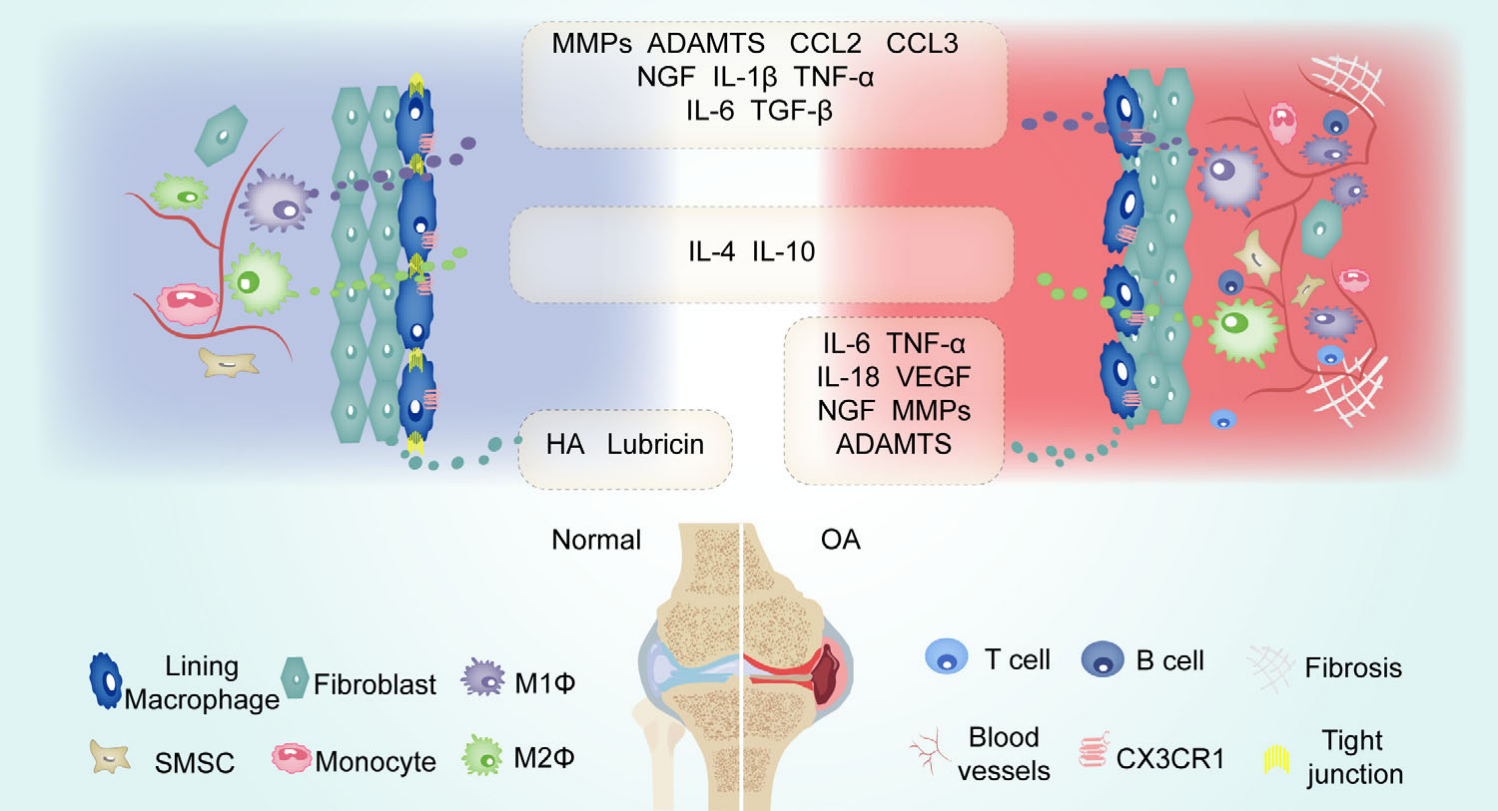
Normal and OA synovium structure, cellular composition and biological functions of synovial cells. The synovium is divided into two layers, the sublining layer (outer layer) and the lining layer (inner layer). The normal synovial lining layer is mainly composed of fibroblasts and macrophages, of which CX3CR1+ macrophages physically isolate the synovium from the joint cavity by tight junctions, forming an immune barrier, with M2 macrophages predominantly secreting IL‐4 and IL‐10, which are involved in anti‐inflammation. Fibroblasts secrete HA and lubricin, which are involved in joint lubrication. The sublining layer is relatively cell‐free, and the cell types are mainly fibroblasts, SMSCs and a few immune cells. The OA synovial lining layer lost its barrier function due to disruption of the tight junctions between CX3CR1+ macrophages. The lining layer is heavily proliferated by fibroblasts, sublining layer is infiltrated by macrophages (M1 predominant), T cells and B cells, with fibrosis and stromal vascularization. MMPs and ADAMTS secreted by fibroblasts and M1 macrophages are associated with cartilage damage, CCL2, CCL3, IL‐1β, IL‐6, TNF‐α, IL‐18, VEGF, TGF‐β with synovitis, synovial angiogenesis and fibrosis, and NGF with pain. NGF (nerve growth factor), MMPs (matrix metalloproteinases), ADAMTS (a disintegrin and metalloproteinase with thrombospondin motifs), CCL2/CCL3 (chemokine (C‐C motif) ligands 2/3), VEGF (vascular endothelial growth factor), TNF (tumor necrosis factor), TGF (tissue grow factor), HA (hyaluronic acid), IL (interleukin).

The occurrence and progression of OA are dominated by cartilage and subchondral lesions. However, the synovial fluid and synovium, as important structures of the joint, are also closely associated with the development of OA. In current clinical OA management, surgeons often only focus on articular cartilage and subchondral bone lesions, while the role of synovial cells and synovial fluid in the OA process are overlooked. Synovial cells could produce significant amounts of pro‐inflammatory factors, inflammatory mediators, and metalloproteinases, resulting in joint inflammation and cartilage destruction (Figure 1).^[^ ^6^, ^7^ ^]^ These inflammatory factors also contribute to synovial angiogenesis, which further promotes immune cell infiltration and aggravates inflammation.^[^ ^8^ ^]^ Moreover, synovial fibroblasts, one of the major populations of synovial cells, can be activated and differentiate into myofibroblast‐like cells, which have increased contractility and lead to synovial fibrosis by depositing excess extracellular matrix (ECM) components.^[^ ^9^ ^]^ In fact, synovial hyperplasia and fibrosis are commonly associated with joint pain and loss of motion.^[^ ^10^ ^]^ On the contrary, recently discovered synovial mesenchymal stem cells (SMSCs), another subpopulation of the synovial cell population, possess stem cell characteristics, including cartilage differentiation potential. More importantly, SMSCs have demonstrated significant potential to mediate inflammation and promote cartilage repair.^[^ ^11^ ^]^ Therefore, they have attracted attention as modalities for cartilage repair. Taken together, synovial cells are the key contributors in OA development and management. In addition, due to the development of nanotherapeutic platforms, from nanoparticles to two‐dimensional nanomaterials and nanoscaffolds, the efficacy of treating joint diseases has been significantly improved.^[^ ^12^ ^]^ Therefore, synovial cell‐based combined with tissue engineering, nanotechnology, and multifunctional platforms have been rationally designed for application in the treatment of OA, which may be the new direction of OA treatment.

Apart from synovial cells, the articular synovial fluid secreted primarily by synovial cells, including hyaluronic acid (HA) and lubricating hormones, also exists in the joint, contributing to joint lubrication.^[^ ^13^ ^]^ Synovial fluid mediates the interactions between tissues within the joint.^[^ ^14^, ^15^ ^]^ Significant changes in the volume, composition and function of synovial fluid after injury or during the progression of the disease cause a range of symptoms, such as pain and swelling. Due to changes that may occur in the intra‐articular tissues as a result of injury and disease, these changes may manifest through the synovial fluid.^[^ ^16^ ^]^ Therefore, synovial fluid can be potentially utilized for the early diagnosis of joint disease.^[^ ^17^, ^18^, ^19^ ^]^

Since the importance of the synovial cells and synovial fluid in maintaining joint health and their role in OA development are recognized, a comprehensive and systematic review of this topic is timely and beneficial. Therefore, this paper firstly discusses the biological functions of the synovium, synovial fluid and synovial cells. The pathological alterations of the synovium and synovial cells in OA are further summarized. More importantly, synovial cell‐related techniques for OA treatment are demonstrated using the latest preclinical and clinical cases. However further studies investigating synovial cells are required to to enhance our understanding of their role in the development and progression of OA. We hope this article will contribute to the future development of synovial cell‐based therapeutic strategies for clinical OA management.

## 2 BIOLOGICAL FUNCTION OF THE SYNOVIUM AND SYNOVIAL FLUID

### 2.1 Synovium

Synovium, also known as the synovial membrane, is located within the synovial joint, lining the inner surface of the joint capsule and can be divided into two layers, namely the sublining layer (outer layer) and the lining layer (inner layer) (Figure 1). The lining layer is mainly composed of fibroblasts and macrophages, while the sublining layer is relatively cell‐free and enriched in type I collagen and microvessels, with lymphatic vessels and nerve fibers (Figure 1).^[^ ^20^ ^]^ The histology of OA synovium shows fibroblast proliferation in lining layer, while the sublining layer is highly infiltrated by immune cells, fibrosis and stromal vascularization (Figure 1).^[^ ^21^ ^]^

The synovium is the primary barrier for molecular exchange between the articular cartilage and plasma. The blood‐joint barrier is also considered to be a series of dual barrier structures, including a synovial interstitium that restricts the diffusion of small molecules and a microvascular endothelium that restricts protein transport.^[^ ^22^ ^]^ A previous study has identified a membrane‐like structure consisting of a specific CX3CR1+ tissue‐resident synovial macrophage population that forms an internal immune barrier in the synovial lining, actively involving in joint inflammation (Figure 1). Thus, synovium also contributes to the mediation of the inflammatory response.^[^ ^23^ ^]^

### 2.2 Synovial fluid

Synovial fluid is an adhesive plasma ultrafiltrate secreted by cells, such as synovial cells and chondrocytes, having lubricating, metabolic and regulatory functions.^[^ ^16^ ^]^ Macromolecules in synovial fluid, including HA, lubricin and phospholipids, are key components of the cartilage surface. Those macromolecules serve an essential role in the lubrication of the cartilage interface.^[^ ^24^ ^]^ Additionally, the synovial fluid contains abundant soluble molecules, including growth factors, cytokines and metalloproteinases secreted by synovial cells or chondrocytes, contributing to intra‐articular cell‐to‐cell communication.^[^ ^25^ ^]^ Changes in the composition or properties of the synovial fluid are related to synovial permeability. For instance, in a healthy state, high molecular weight molecules such as lubricin and HA do not penetrate easily, while small molecules such as growth factors and cytokines diffuse out via the synovium. When the synovium is in an inflammatory or hyperplastic state, the permeability of the synovium changes, leading to a decrease in the concentration of HA and lubricin in synovial fluid.^[^ ^26^ ^]^ Studies have shown the difference in the metabolomic profile of synovial fluid in normal, early as well as late‐stage OA patients. For example, changes in degradation of N‐glycan and metabolism of silicic acid in synovial fluid during early and late OA were observed.^[^ ^27^ ^]^ Since N‐glycan and sialic acid are essential components of lubricin, the degradation and reduction in both molecules indicate an impaired function of synovial fluid, therefore, metabolomic phenotypic changes in synovial fluid may reflect overall disease progression.^[^ ^27^ ^]^ In fact, synovial fluid has been used for clinical diagnosis. For instance, Fu et al. showed that synovial fluid viscosity levels were significantly lower in patients with periprosthetic infection (7.93 mPa s, 3.0–15.0) than in patients with aseptic loosening of the prosthesis (13.11 mPa s, 6.3–20.4), and further found that synovial fluid viscosity was superior to CRP, ESR, and d‐dimer with a sensitivity of 93.33% and specificity of 66.67%, suggesting that it is a promising method for diagnosing periprosthetic infections.^[^ ^28^ ^]^ In another clinical study (48 knees), a large number of synovial fluid biomarkers were characterized using a high‐sensitivity multiplex immunoassay. Vascular cell adhesion molecule‐1 (VCAM‐1), MMP‐3, intercellular adhesion molecule 1 (ICAM‐1), tissue inhibitor of metalloproteinases 1 (TIMP‐1), VEGF and monocyte chemotactic protein 1 (MCP‐1) were confirmed to associate with synovial fluid neutrophil (elastase) and/or macrophage (CD14 and CD163)‐specific markers that has been used to predict knee OA progression, indicating those new biomarkers could reflect macrophage and neutrophil‐mediated inflammation.^[^ ^29^ ^]^

### 2.3 The microenvironment of the synovium

Schofield introduced the concept of the “microenvironment” in 1978, a dynamic growth environment composed of various cells, external matrix nutrients and growth factors facilitating the survival and functions of cells.^[^ ^30^ ^]^ The primary strutures of the synovial microenvironment are made up of stromal cells, ECM molecules, and other resident tissue cells ^[^ ^31^ ^]^ Fibroblasts and macrophages, which are the main stromal cells in OA, secrete large amounts of inflammatory factors (IL‐1β and IL‐6, TNF‐α). These factors promote synovial inflammation and may lead to cartilage lesions.^[^ ^32^ ^]^ In addition, abnormal signals from the ECM can trigger chronic inflammation in the OA synovium. For instance, the over‐deposition of ECM molecules in fibroblasts is a primary characteristic of synovial fibrosis.^[^ ^10^ ^]^ Hypoxia and inflammation are also closely related, where the synovial hypoxic environment can promote synovial fibrosis and vascularization, further accelerating OA progression.^[^ ^33^ ^]^ Therefore, alterations in the synovial microenvironment are crucial for the pathological progression of OA. Recently, Canavan et al. arthroscopically obtained synovial tissue and used explant‐conditioned media to mimic the synovial microenvironment in vitro via co‐culture with monocyte‐derived dendritic cells (DCs).^[^ ^34^ ^]^ Their results showed that the microenvironment promotes the secretion of inflammatory cytokines, chemokines and adhesion molecules in DCs, induces DCs maturation as well as metabolic changes (glycolysis).^[^ ^34^ ^]^ The study also confirmed the interaction between synovial tissue and immune cells .^[^ ^34^ ^]^

## 3 BIOLOGICAL FUNCTION OF SYNOVIAL CELLS

### 3.1 Synovial fibroblasts and macrophages

Synovial macrophages and synovial fibroblasts, which constitute the majority of synovial cells, have recently ben considered to play a crucial role in the onset and progression of OA .^[^ ^35^, ^36^ ^]^ In a mouse model of arthritis, two different fibroblast subpopulations have been identified based on single‐cell transcriptional analysis. FAPα+THY1+ synovial fibroblasts located in the sub‐synovial layer induce joint inflammation with minimal impact on bone and cartilage. FAPα+ THY1‐ synovial fibroblasts, on the other hand, are confined to the inner synovial layer, causing bone and cartilage damage without eliciting inflammation.^[^ ^37^ ^]^ Chou et al. sequenced human OA synovial tissue and found that synovial subendothelial fibroblasts express collagen and stromal cell‐derived factor 1 (CXCL12) genes for the synthesis of ECM components. Synovial endothelial fibroblasts mainly express genes that produce essential components of synovial fluid, including lubricin (PRG4) and HA.^[^ ^25^ ^]^ In the inflammatory state, activation of the NF‐κB pathway by activating the cell surface receptor Toll‐like receptor (TLR) induces fibroblasts to secrete various inflammatory factors, including IL‐6, VEGF, and matrix metalloproteinases (MMP‐1 and MMP‐3), which further aggravates synovial inflammation and cartilage destruction.^[^ ^38^ ^]^ Apart from the NF‐κB pathway, the Wnt/β‐catenin pathway is also activated in synovial fibroblasts in the inflammatory state, facilitating fibroblast proliferation and the secretion of inflammatory factors and catabolic enzymes.^[^ ^39^, ^40^ ^]^ In addition, NGF is highly expressed in OA synovial fibroblasts and macrophages, contributing to joint pain.^[^ ^41^ ^]^ For instance, in OA patients who underwent total hip arthroplasty, NGF was mainly expressed in synovial fibroblasts, and synovial macrophages with high CD14 expression also expressed higher levels of NGF. More importantly, analysis of clinical data showed a negative relationship between JOA hip pain scores and synovial NGF mRNA expression levels (*r* = −0.337, *P* = 0.017) and a positive relationship between CSI scores and synovial NGF mRNA expression levels in OA patients (*r* = 0.358, *P* = 0.011). As a result, the level of NGF expression in synovial cells is closely related to pain and central sensitization (CS) in OA patients.^[^ ^41^ ^]^ Furthermore, a recent single‐cell RNA sequencing study revealed that subpopulations and transcriptomes of synovial fibroblasts in painful and non‐painful areas of knee OA patients were different, further indicating that fibroblasts in early painful areas promote fibrosis, inflammation, and fibroblasts in early OA and end‐stage pain areas promote neuronal growth and activate injury‐sensing signaling pathway.^[^ ^42^ ^]^

Activated synovial macrophages can be divided into two polarized forms. The M1 macrophages (pro‐inflammatory status) express the predominant markers CD11c, MHCII and CD86 receptors and produce several pro‐inflammatory cytokines (IL‐1β, TNF‐α). On the other hand, the M2 macrophages (pro‐healing status) release anti‐inflammatory cytokines (IL‐10) and express the scavenger receptor CD163 and the mannose receptor CD206.^[^ ^43^, ^44^ ^]^ M1 macrophages have been shown to be induced by interferon‐γ, lipopolysaccharide (LPS) or TNF‐α, and secrete a large number of pro‐inflammatory cytokines, such as TNF‐α, IL‐1, 6 and 12,^[^ ^45^ ^]^ while M2 macrophages can be induced by cytokines IL‐4 or IL‐13, and secrete IL‐10 to participate in anti‐inflammatory effects.^[^ ^46^ ^]^ IFN‐γ induces STAT1 activation via the JAK/STAT pathway and promotes M1 macrophage formation,^[^ ^47^ ^]^ while IL‐4 or IL‐13 promote M2 macrophage formation via STAT3 and STAT6.^[^ ^48^ ^]^ In addition, hypoxia is another factor contributing to macrophage polarization, where HIF‐1α (hypoxia‐inducible factor) promotes M1 macrophage formation and HIF‐2α induces M2 macrophage polarization.^[^ ^49^ ^]^ Studies also have shown that squid type II collagen and quercetin can promote macrophage polarization toward M2 type in a rat model of OA, thereby suppressing inflammation and promoting cartilage repair.^[^ ^50^, ^51^ ^]^ Therefore, cytokines, signaling pathways, transcription factors and tissue environment are all related to the phenotypic regulatory mechanisms of macrophages. A recent study compared synovium from OA patients and healthy donors, the proportion of M1/M2 macrophages was substantially higher in OA patients than in the healthy group (greater than twofold, *p* < 0.01) and was positively related to the K‐L score.^[^ ^52^ ^]^ Therefore, the regulatory mechanism of macrophage polarization morphology may be a new target for the treatment of OA and needs to be further investigated, especially in the field of OA .

Interestingly, an increasing number of studies suggest that M1 and M2 represent only two extreme phenotypes of macrophage activation that cannot fully reflect the complex tissue environment.^[^ ^53^, ^54^ ^]^ Wood et al. identified two distinct subpopulations of macrophages that express certain genes associated with both M1 and M2 by RNA‐Seq sequencing, suggesting that the activation state of these macrophages lies between the M1 and M2.^[^ ^55^ ^]^ Therefore, a comprehensive analysis of the cell type, developmental origin and function of macrophages in healthy and inflamed joints is required.

### 3.2 The potential origin and expression of SMSCs

SMSCs were first isolated from the human knee synovium in 2001. Research has shown that SMSCs have stem cell properties and multidirectional differentiation capabilities similar to other mesenchymal stem cell sources.^[^ ^56^ ^]^ However, SMSCs were found to have similar characteristic lamellar bodies (LBs) as synovial fibroblasts, suggesting that SMSCs may originate from the synovial lining.^[^ ^57^ ^]^ Also, synovial fibroblasts and SMSCs exhibited similar spindle morphology in vitro.^[^ ^58^, ^59^ ^]^ In addition, markers of synovial fibroblasts (VCAM‐1, CD44 and β 1 integrin) are expressed on the surface of SMSC.^[^ ^56^ ^]^ Several experiments have demonstrated that fibroblasts possess osteogenic, chondrogenic and adipogenic abilities.^[^ ^60^, ^61^ ^]^ Taken together, these studies suggest that SMSCs and synovial fibroblasts cannot be clearly distinguished. Therefore, the new hypothesis that SMSCs are immature fibroblasts has gained popularity. In other words, synovial fibroblasts may be derived from SMSCs. Apart from synovium, SMSCs were also identified in other sites. In fact, the origin of SMSCs is currently inconclusive. Li et al. found in a rheumatoid animal model that normal synovial fibroblasts contained small amounts of mesenchymal stem cells from bone marrow, while in the inflammatory state a large number of bone marrow‐derived MSCs influx into the synovium and further differentiated into synovial fibroblasts. As a result, SMSCs may originate from the bone marrow.^[^ ^62^ ^]^ Another study compared the properties of MSCs from the surface, stromal and perivascular areas of the synovium, demonstrating that MSCs from the perivascular areas possess the best proliferative and chondrogenic capacity, hypothesizing that SMSCs may be derived from the perivascular area.^[^ ^63^ ^]^ In addition, a recent study found that Gdf5 lineage cells from mice possess joint progenitor cell activity and are also observed in adult synovium. These cells are derived from the embryonic interarticular zone and are located in the synovial lining. More importantly, Gdf5 lineage cells showed the capacity for proliferation as well as cartilage differentiation after cartilage injury, suggesting that SMSCs may be derived from the embryonic interarticular zone.^[^ ^64^ ^]^ Subsequent evidence has confirmed that SMSCs express CD44, CD73, CD105, CD90, CD106 (VCAM‐1) and STRO‐1, with the limited expression of CD45, CD34, CD14 and HLA‐DR.^[^ ^65^, ^66^ ^]^

### 3.3 Paracrine effects, intercellular contact, and immunomodulatory capacity of MSCs

Paracrine signaling is the main mode of extracellular communication between cells. It has been known that MSCs can secrete various paracrine factors, such as cytokines, growth factors and extracellular vesicles (EVs).^[^ ^67^ ^]^ Although the paracrine factors secreted from different types of MSCs may vary, those factors have a similar regenerative capacity, and in particular, they can promote cartilage regeneration.^[^ ^68^, ^69^ ^]^ Interestingly, a recent study has shown that the proteins secreted by the bone marrow, adipose tissue, and synovial mesenchymal stem cells (BMSCs, AMSCs, and SMSCs) in different environments differ in type and expression level, and found that inflammatory conditions promote the secretion of paracrine factors associated with cartilage regeneration(anti‐inflammatory and anti‐chondrocyte apoptosis), indicating the therapeutic potential of MSCs in cartilage repair.^[^ ^70^ ^]^

**FIGURE 2:**
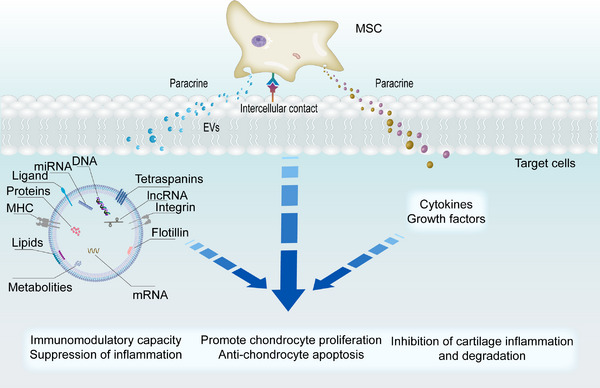
MSC is involved in joint inflammation and cartilage repair through multiple mechanisms. MSC regulates immune cells (T cells, B cells, macrophages) by secreting paracrine factors (cytokines, growth factors and EVs), and intercellular contacts to exert immunomodulatory functions and reduce inflammation, such as inhibiting T and B cell proliferation and inducing macrophage polarization. MSC promotes chondrocyte proliferation, migration and anti‐apoptosis by secreting EVs, and inhibit chondrocyte inflammatory factor release and cartilage degradation by secreting paracrine factors and intercellular contacts.

In addition to promoting cartilage regeneration, the secreted paracrine signals and direct cell–cell contact also possess the immunomodulation capacity via mediating intercellular communication (Figure 2). In a mouse abdominal sepsis model, MSC‐secreted TGF‐β was found to promote LPS‐induced polarization of MI macrophages toward M2, thereby reducing inflammation.^[^ ^71^ ^]^ Another study showed that MSC are involved in anti‐inflammatory by secreting PGE 2 to promote M2 macrophage polarization and increase IL‐10 release levels.^[^ ^72^ ^]^ The mechanism for MSC‐induced macrophage polarization is unclear and may be related to the soluble factors secreted by MSC. In addition to macrophages, MSCs can also regulate T cells. MSCs inhibit T cell proliferation and IFN‐γ release, upregulate the expression of the anti‐inflammatory factor IL‐4, and exhibit immunosuppressive properties.^[^ ^73^ ^]^ MSCs also inhibit the proliferation, activation, and differentiation of CD4+ T cells, while producing immunosuppression associated with IL‐10‐mediated secretion.^[^ ^74^ ^]^ In addition to paracrine mechanisms, the direct intercellular contact between MSC and T cells is also involved in immunomodulatory processes. Activation of TLRs on MSCs increases the induction of Treg through Notch signaling and exerts immunomodulatory effects depending on direct cell‐to‐cell contact.^[^ ^75^ ^]^ In addition, Akiyama et al. showed that BMSCs induce T cell apoptosis via the Fas/FasL pathway.^[^ ^76^ ^]^ MSCs can also inhibit a range of biological behaviors of B cells, such as proliferation, plasma cell differentiation and maturation, as well as antibody production. For instance, Nan et al. isolated B cells from mouse spleen tissue co‐cultured with MSCs and showed inhibition of B cell differentiation and reduced IgM and IgG production.^[^ ^77^ ^]^ Another study showed that BMSCs inhibit antigen‐dependent proliferation and differentiation of B cells to plasma cells. This process is mediated by intercellular contacts of the programmed death 1 (PD‐1)/PD ligand pathway.^[^ ^78^ ^]^

Apart from direct modulation of immune cells, MSCs also regulate the pro‐inflammatory factor released from other cells, indirectly mediating the inflammation. For example, a recent study used an LPS‐stimulated chondrocyte model to assess the anti‐inflammatory efficacy of MSCs. The results showed that MSCs suppressed chondrocyte inflammation by down‐regulating chondrocyte expression of the inflammatory genes through paracrine and intercellular contacts.^[^ ^79^ ^]^ A similar study demonstrated that co‐culture of SMSCs with chondrocytes down‐regulated the expression of MMP‐13 and up‐regulated the expression of COL2A1, Aggrecan, while injection of SMSCs into the joints of OA rats suppressed the secretion of inflammatory factors and increased the expression of COL2A1, Aggrecan.^[^ ^80^ ^]^

In addition, EVs secreted by MSCs represent another paracrine mechanism which plays an important role in reducing inflammation and cartilage damage (Figure 2). EVs from MSCs contain many bioactive components, such as microRNAs and proteins, which are transferred to target cells via the paracrine pathway, providing an immunosuppressive effect. Researchers injected exosomes from BMSCs into the joint cavity and showed that the exosomes promoted the polarization of synovial macrophages from M1 to M2, reduced inflammation and cartilage damage, and prevented the progression of OA.^[^ ^81^ ^]^ Another study showed that EVs from MSCs exhibit immunosuppressive capacity and exert immunomodulatory effects on inflammatory arthritis by inhibiting T cell proliferation and inducing Treg populations.^[^ ^82^ ^]^ Wang et al. found that miR‐31 within SMSC‐EVs upregulated E2F1 and PTTG1 by targeting and inhibiting KDM2A, significantly reduced the levels of IL‐1β, IL‐6, promoted chondrocyte proliferation and migration and resulted in a reduction of inflammation and cartilage damage.^[^ ^66^ ^]^

In conclusion, MSCs may offer a promising treatment for OA in inflammation suppression and cartilage repair due to their paracrine effect and immunomodulatory capacity. That being said, the therapeutic efficiency of different MSC, and EV therapy, remain elusive, and SMSCs should be investigated thoroughly prior to the full clinical translation.

### 3.4 Cartilage differentiation potential and comparison with other MSCs

As common progenitor cells exist between the synovium and cartilage, SMSCs express higher levels of CD44 (HA receptor) and also express uridine diphosphate glucose dehydrogenase, a key enzyme for HA synthesis.^[^ ^83^ ^]^ Hence, SMSCs potentially have an improved chondrogenic capacity than other stem cells. Several studies have shown that SMSCs have enhanced cartilage differentiation potential than BMSCs.^[^ ^84^, ^85^ ^]^ Interestingly, a recent study compared the chondrogenesis of different MSCs (bone marrow, synovium, synovial fluid) in donors with late‐stage OA who underwent arthroplasty, concluded that BMSC had the best chondrogenic differentiation capacity with elevated collagen II production.^[^ ^86^ ^]^ That being said, SMSCs from this particular study were from the diseased joint cavity, while BMSCs are extra‐articular, so the difference in pathological anatomical location may affect the results. Another study comparing the chondrogenic differentiation potential of SMSCs and BMSCs derived from equine found no advantage for SMSCs.^[^ ^87^ ^]^ Consequently, the comparison of chondrogenic potential between SMSCs and BMSCs remains inconclusive due to both the species and pathological difference.

Often, the differentiation and endogenous regenerative capacity of BMSCs decreases with increasing donor age, while the differentiation capacity of AMSCs is associated with obesity.^[^ ^88^ ^]^ However, the differentiation capacity of SMSCs was less affected by age or obesity.^[^ ^88^ ^]^ For example, researchers collected fibers, adipose synovium, and subcutaneous fat from young and old donors and found that MSCs derived from synovium produced more cartilage matrix than those derived from fat. Furthermore, few differences differentiation potential were observed between young and old donors.^[^ ^65^ ^]^ In addition, it has been shown that adult SMSCs from different age groups can be passed in vitro up to 10 generations with minimal cellular senescence.^[^ ^56^ ^]^ Therefore, SMSCs may hold great clinical potentials. It should be noted that the origins of the SMSCs (such as knee and hip) also determine the chondrogenic differentiation capacity of the SMSCs. For example, Hatakeyama et al. demonstrated that SMSCs from the knee showed greater potential and differentiation levels than SMSCs from the hip joint.^[^ ^89^ ^]^ Hence, the origins of SMSCs need to be considered during the clinical treatment.

## 4 INTERACTIONS BETWEEN SYNOVIAL CELLS AND CHONDROCYTES

The joint cavity is a closed cavity that is composed of different tissues. Synovium and cartilage are the primary tissues in the joint, and communications between them are essential to maintain the intra‐articular microenvironment and joint homeostasis. Abnormal communication induces the development of joint disease. For instance, activated synovial fibroblasts and chondrocytes produce large amounts of MMPs. Synovial fibroblasts, on the other hand, apart from secreting MMPs, also secreting pro‐inflammatory cytokines (IL‐1β, TNF‐α, IL‐6).^[^ ^90^ ^]^ In addition, activated synovial macrophages can also release various pro‐inflammatory mediators (e.g., TNF‐α, IL‐1β, IL‐6).^[^ ^91^ ^]^ Often, these pro‐inflammatory mediators can further promote synovial fibroblasts to secrete MMPs and ADAMTS, resulting in the accelerated degradation of cartilage ECM.^[^ ^43^ ^]^ Single‐cell RNA sequencing showed that in OA patients, cytokines (TNF, IL‐6, IL‐1β) are mainly secreted by synovial fibroblasts and macrophages, which act as upstream regulators and lead to chondrocyte expression of cartilage degradation‐related genes.^[^ ^25^ ^]^ Within the chondrocytes, the accumulation of excessive inflammatory secretions can cause mitochondrial DNA damage and the over‐production of reactive oxygen species (ROS), which contributes to increased catabolism within chondrocytes, ultimately leading to an imbalance between cartilage repair and destruction.^[^ ^92^ ^]^

In addition to cytokines, EV‐mediated paracrine effects between synovial cells and chondrocytes represent another way of intercellular communication. For example, researchers found that IL‐1β‐stimulated synovial fibroblasts increased the number of exosomes, and fifty miRNAs were identified to be differentially expressed, compared to unstimulated fibroblasts in in vivo and in vitro experiments. The miRNA‐containing exosomes resulted in altered articular chondrocyte phenotype, increased the expression of cartilage matrix degradation genes (MMP‐13, ADAMTS‐5) and decreased the expression of cartilage matrix synthesis genes (COL2A1, ACAN), ultimately promoting cartilage degeneration and pathological progression of OA.^[^ ^93^ ^]^ Moreover, exosomes from chondrocytes can mediate communication between chondrocytes and macrophages. Exosomes from OA chondrocytes inhibit ATG4B expression mediated by microR‐449a‐5p, increase IL‐1β production in macrophages, and further aggravate synovitis.^[^ ^94^ ^]^

**FIGURE 3:**
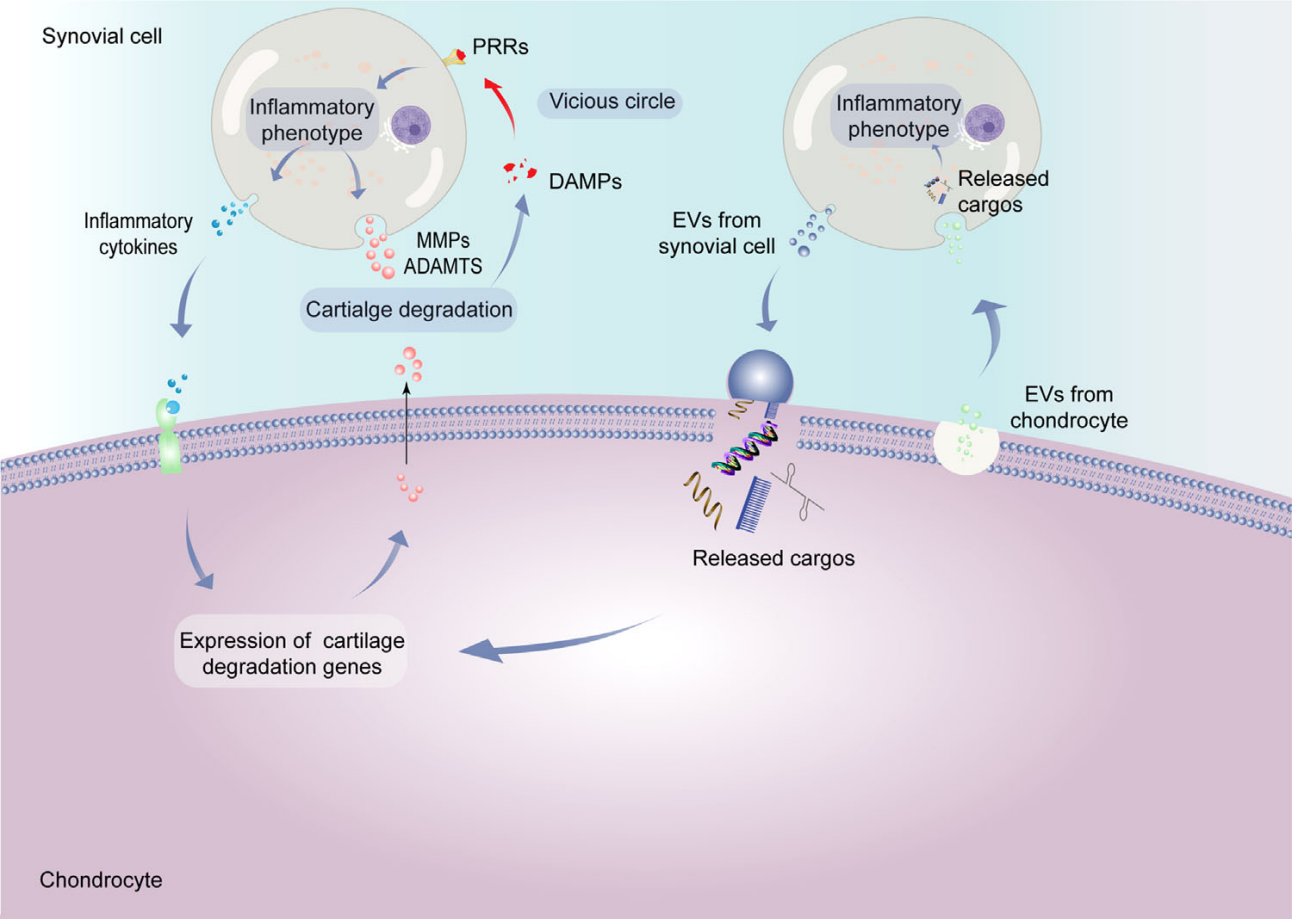
Mechanisms of synovial cells and chondrocytes communication in the pathological progression of OA. Cytokines (e.g., IL‐6, TNF‐α, IL‐1β) are secreted by synovial cells (fibroblasts and macrophages) into the synovial fluid and act as upstream regulators, causing changes in chondrocyte phenotype and leading to cartilage degradation. Synovial cells can also secrete MMPs and ADAMTs directly, leading to cartilage degradation. EVs from synovial cells and chondrocytes interact by carrying proteins, RNA, and other cargoes, resulting in altered chondrocyte and synovial cell phenotypes, leading to expression of cartilage degradation genes and secretion of inflammatory cytokines. Cartilage degradation products such as cartilage fragments, crystals and extracellular matrix degradation molecules constitute DAMPs that activate synovial cell surface pattern receptors (PRRs), prompting synovial cells to express inflammatory phenotypes and secrete cytokines, a vicious cycle is formed. Together, these mechanisms contribute to the pathological progression of OA.

To this end, phenotypic changes in synovial cells and chondrocyte mediated by cytokines contribute to the progression of OA. At the same time, EV communication mechanism between synovial cells and chondrocytes is another communication mechanism that plays a significant role in the pathological changes of OA (Figure 3).

## 5 THE PATHOLOGICAL CHANGE OF SYNOVIAL CELLS IN OA

### 5.1 Synovitis

OA has been considered a “wear and tear” joint disease for a long time in the past, however, it is now considered by many to be a low‐grade chronic inflammatory disease.^[^ ^95^ ^]^ Meanwhile, synovitis as synovial inflammation is a typical characteristic of inflammatory arthritis, which contributes to OA development. The histological changes observed in the synovium from OA donors typically include inflammatory “synovitis” features, including proliferation of the synovial lining as well as a large infiltration of immune cells.^[^ ^96^ ^]^ Compared with patients suffering from rheumatoid arthritis, the degree of inflammatory cell infiltration in the synovial tissue is much lower in patients with OA.^[^ ^97^ ^]^ However, the mechanism that triggers synovitis remains unknown. The innate immune system, as the first line of defense in disease development, is triggered by pathogen‐associated molecular patterns (PAMPs) and danger‐associated molecular patterns (DAMPs) binding to pattern recognition receptors (PRRs)^[^ ^98^ ^]^ and is thought to be associated with the initiation mechanism of synovitis (Figure 3). Joint injury induces the release of DAMPs‐related molecules, such as cartilage matrix catabolic products, plasma proteins, intracellular alarmins and crystals.^[^ ^99^ ^]^ These factors further promote the local secretion of inflammatory mediators by activating PRRs on chondrocytes, macrophages and fibroblasts, thereby promoting cartilage degeneration and synovitis.^[^ ^99^ ^]^ Often, PRRs include TLRs and receptors for advanced glycosylation end products (RAGE).^[^ ^100^ ^]^ All of the identified human functional TLRs, except for TLR3, are known to activate transcription factors via MYD88‐dependent signaling pathways, thus producing cytokines and chemokines.^[^ ^101^ ^]^ The interaction of RAGE with its ligands causes the upregulation of molecules, including cytokines, adhesion molecules and MMPs, associated with inflammatory responses, contributing to the localized innate immunity involved in the OA pathogenesis.^[^ ^102^ ^]^ In addition, the complement system plays a role in another innate immune mechanism. Its activation is likely to be associated with OA synovitis and cartilage damage. Previously, researchers found that in the osteoarthritis group, patients had significantly higher levels of complement components (C4d, C3bBbP and sTCC) than in the normal group, median levels increased by fourfold, twofold and fourfold, respectively.^[^ ^103^ ^]^ Furthermore, molecules of the cartilage ECM, including fibronectin, aggrecan and cartilage oligomeric matrix protein (COMP), have been proven to activate the complement system to exacerbate synovial inflammation.^[^ ^104^, ^105^, ^106^ ^]^

A previous study showed that early‐stage OA patients have significantly higher histological synovitis scores than those with advanced OA, and patients with high synovitis scores presented more often with symptoms, such as resting pain.^[^ ^107^ ^]^ Therefore, synovitis scores do not directly reflect the severity of joint degeneration, but the impact of synovitis on OA progression cannot be overlooked. Liao et al. demonstrated in a trauma‐induced mouse model of OA that acute synovitis precedes the onset of articular cartilage degeneration.^[^ ^108^ ^]^ Namely, synovitis often appears early in the disease, even before cartilage damage, thus synovitis may be a contributing factor to cartilage damage. In a cohort study investigating the relationship between changes in synovitis on contrast‐enhanced MRI (CE‐MRI) and cartilage degeneration in patients with knee OA over 2 years, the total synovitis score increased on average in patients with cartilage deterioration, while the total synovitis score decreased on average in patients without cartilage deterioration.^[^ ^109^ ^]^ In a prospective and multicenter study that included 531 patients over three years, ultrasound detection of knee effusion was shown to predict pain levels, imaging progression, and joint replacement surgery.^[^ ^110^ ^]^ A recent randomized multicenter controlled trial showed a positive correlation between medial peri‐meniscal synovitis and knee pain as measured by CE‐MRI, while suprapatellar synovitis and medial peri‐meniscal synovitis were positively correlated with knee function.^[^ ^111^ ^]^ These studies suggest that synovitis may be associated with OA clinical symptoms, disease progression and joint function. Therefore, suppression of synovitis may be a future intervention for the treatment of OA.

### 5.2 Angiogenesis

**FIGURE 4:**
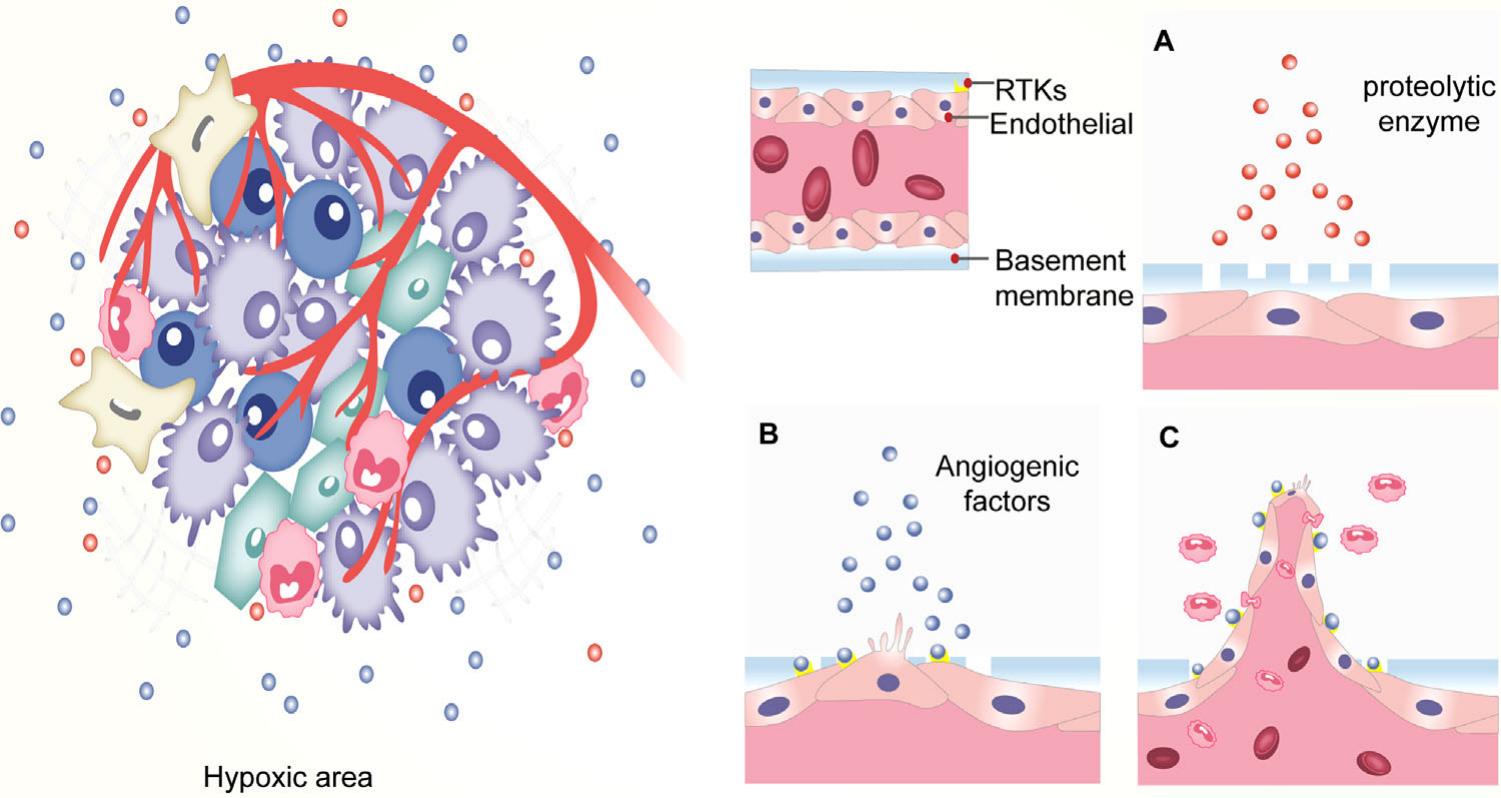
Synovial cells are involved in the process of synovial angiogenesis. In the OA inflammatory environment, synovial macrophages and fibroblasts secrete cytokines such as IL‐6, IL‐8, TNF‐α, which induce the production of growth factors such as VEGF and FGF by macrophages and fibroblasts in the synovial hypoxic environment, and also regulate the secretion of chemokines, MMPs, adhesion molecules, and participate in endothelial cell activation, basement membrane degradation, endothelial cell proliferation and migration, and tubular lumen formation, further promote inflammatory cell infiltration. These factors secreted by synovial cells are called angiogenic factors.

Synovitis is present throughout the course of OA that is highly related to the onset and progression of OA.^[^ ^112^ ^]^ Angiogenesis and synovitis are two related processes, as synovitis stimulates angiogenesis and vice versa.^[^ ^113^ ^]^ Angiogenesis is a process of forming new blood vessels by sprouting on the basis of the original vascular endothelium, and the new vessels promote inflammatory cell infiltration and increase synovial permeability to macromolecules, further promoting synovitis. The angiogenic process consists of many steps that are mediated by a range of different mediators, including growth factors (HIF and VEGF), pro‐inflammatory cytokines, chemokines, proteases, matrix components and cell adhesion molecules (Figure 4).^[^ ^8^, ^114^ ^]^ The angiogenic process begins with growth factors secreted by cells in the synovium. Released VEGF and fibroblast growth factor (FGF), which bind to receptor tyrosine kinase (RTKs) in endothelial cells (ECs), activate ECs to release proteolytic enzymes that degrade the endothelial basement membrane and perivascular extracellular matrix (Figure 4).^[^ ^114^ ^]^ This process leads to the migration and further proliferation of ECs into the interstitial tissue, forming lumens, then a new basement membrane is synthesized, and finally, capillaries are formed.^[^ ^115^ ^]^ HIF‐a, a key factor in angiogenesis, is significantly elevated in hypoxic environments, and activates ECs by inducing VEGF and FGF production in synovial cells, thereby initiating the angiogenic process.^[^ ^116^ ^]^ Hypoxia is consequently considered to be a factor promoting synovial angiogenesis, and it has been demonstrated in several experiments that hypoxia promotes synovial angiogenesis.^[^ ^116^, ^117^ ^]^ In addition, synovial angiogenesis is also closely related to chronic synovitis, with angiogenesis promoting the acute to chronic inflammatory transition that occurs in all stages of OA.^[^ ^113^, ^118^ ^]^

### 5.3 Synovial fibrosis and hyperplasia

Apart from the inflammatory cell infiltration and new vascular formation, the histological features of OA synovitis include the proliferation and hyperplasia of lining cells.^[^ ^21^ ^]^ Synovial fibrosis exists in different stages of OA, is stimulated by synovitis. For example, IL‐1β is not only a key regulator of TGF‐β, but also stimulates IL‐1β receptors on fibroblasts, leading to fibroblast proliferation and promoting fibrosis,^[^ ^119^ ^]^ and IL‐6 promotes fibrosis by activating the Notch/STAT3 pathway.^[^ ^120^ ^]^ Recent studies have found that synovial fibroblast and macrophage pyroptosis is involved in synovial fibrosis and is associated with the release of inflammatory factors (IL‐1β, IL‐18) due to activated NLRP3 inflammasome.^[^ ^121^, ^122^ ^]^ One of the main features of synovial fibrosis is an imbalance between the synthesis and breakdown of type I collagen in the ECM of fibroblasts, leading to excessive deposition, and over‐proliferation of fibroblasts.^[^ ^123^ ^]^ Synovial thickness was measured using MRI scans after total knee arthroplasty (1.9 mm ± 0.2 mm) and was significantly different in patients diagnosed with fibrosis (4.4 mm ± 0.2 mm) compared to patients without fibrosis (2.5 mm ± 0.4 mm).^[^ ^124^ ^]^ Previous studies have shown that activation of the classical Wnt/β‐catenin pathway induces synovitis. In a mouse model, inhibition of the Wnt/β‐catenin pathway reduces synovial fibroblast proliferation and fibrosis, suggesting not only that the Wnt/β‐catenin pathway can promote fibrosis, but also that synovitis can promote fibrosis.^[^ ^125^ ^]^ The researchers measured OA and non‐OA synovial fluid samples by ELISA and found higher TGF‐β concentrations in OA synovial fluid samples (non‐OA, 58.29 pg −1 mL ± 16.03, OA, 113.4 pg −1 mL ± 19.6; *p* = 0.03).^[^ ^126^ ^]^ Also, TGF‐β secreted by synovial cells is closely related to the fibrotic cascade response. For instance, it has been demonstrated that injection or transfection of TGFβ facilitates synovial proliferation.^[^ ^127^, ^128^ ^]^ Another study confirmed that genes promoting fibrosis (PLOD2, LOX, etc.) were significantly upregulated in TGFβ‐induced synovial fibrosis mice.^[^ ^129^ ^]^ TGF‐β as a pro‐fibrotic factor by binding to the TGF‐β type II receptor via different receptors (ALK5, ALK1) that phosphorylate the receptors Smads, Smad2/3 and Smad1/5/8, respectively.^[^ ^130^ ^]^ In addition, the overexpression of connective tissue growth factor (CTGF) in mouse joints has been reported to cause synovial fibrosis.^[^ ^131^ ^]^ Other factors, including lysine proprotein, 2‐ketoglutarate dioxygenase 2 (PLOD2) and tissue inhibitor of metalloproteinases (TIMP1), also increased in the fibrotic synovium from OA patients.^[^ ^132^ ^]^

Apart from the cytokines, microenvironment—hypoxia may be associated with synovial fibrosis. For instance, researchers found that inhibition of HIF‐1α in OA rats down‐regulated the expression of genes associated with fibrosis (TGF‐β, PLOD2).^[^ ^121^ ^]^ Zhang et al. found elevated expression levels of fibrosis (TGF‐β1, TIMP‐1) and hypoxia (HIF‐1α) markers in rat arthritic synovium, and demonstrated that imperatorin could reduce synovitis and synovial fibrosis by inhibiting HIF‐1α/NLRP3 signaling.^[^ ^133^ ^]^ Since hypoxia is closely related to synovitis, angiogenesis and fibrosis, improving the synovial hypoxic environment may contribute to joint protection.

To this end, synovial fibrosis and hyperplasia contribute to joint stiffness and dysfunction. Studies have confirmed that synovial fibrosis scores are negatively correlated with K‐L scores, thus suggesting that radiological severity may be positively correlated with the degree of synovial hyperplasia.^[^ ^134^ ^]^ Signaling pathways associated with synovial cells in the pathological changes of OA (Table 1).

**TABLE 1:** Signaling pathways associated with synovial cells in the pathological changes of OA.

Signaling pathways	Mechanisms	Ref.
NF‐kB	Promotes the expression of TNFα, IL‐1β, 6 and 8 by synovial fibroblasts	[194]
Wnt/β‐catenin	Promotes synovitis, synovial fibrosis Mediates the production of ADAMTS‐7 and −12 by OA synovial fibroblasts, causing cartilage degradation	[125] [195]
Noth	Promotes VEGF/Ang2‐induced synovial angiogenesis and endothelial cell invasion in arthritis	[196]
mTOR	Induces M1 polarized macrophages to promote hypertrophic chondrocyte differentiation and maturation	[197]
ERK/p38/JNK	Promotes the secretion of IL‐6 and TNF‐α by synovial fibroblasts	[198]
TGF‐β	Promotes fibroblast proliferation and ECM deposition, leading to synovial fibrosis	[145]
TLR	Promotes the expression of inflammatory factors and MMP‐13 by activating the NF‐kB pathway	[199]

## 6 POTENTIAL STRATEGIES FOR SYNOVIAL CELL‐BASED THERAPY FOR OA

### 6.1 Targeted therapy for synovial fibroblast and macrophage

#### 6.1.1 Targeted inhibition of synovial fibroblast and macrophage‐mediated inflammation, angiogenesis, fibrosis, pain and cartilage damage

**FIGURE 5:**
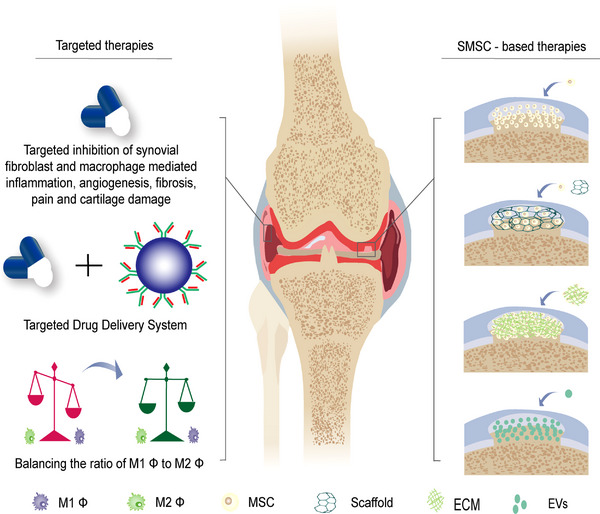
Potential strategies for synovial cell‐based therapy for OA. Targeted therapeutic strategies include targeting synovial fibroblast and macrophage mediated inflammation, angiogenesis, fibrosis, pain and cartilage damage and the development of related targeted drug delivery systems. SMSC‐based therapies mainly include direct SMSC implantation, combined with advanced technologies such as biological scaffolds or scaffold‐free tissue engineering, and SMSC‐derived EV therapy, which mainly promotes cartilage repair and inhibits inflammation.

During the progression of OA, cytokines (IL‐1, IL‐6, TNFα) secreted by synovial cells can further contribute to the secretion of ROS, MMPs and ADAMTS.^[^ ^135^ ^]^ Those synovial cell‐associated products contribute to synovitis, synovial fibrosis, angiogenesis and cartilage destruction, and are closely related to the clinical symptoms of OA, including joint pain, swelling and limited functional activity. As a result, targeted inhibition of the secretion of inflammatory factors, metalloproteinases, and polyproteinases by synovial cells, as well as inhibition of synovial fibrosis and synovial angiogenesis, may offer potential treatments for OA (Figure 5), which have been validated in vivo and in vitro. For example, Ma et al. showed that vanilloid acid (VA) decreased the release of IL‐1β and IL‐18 and effectively reduced the level of NGF in synovial fibroblasts. To assess pain behavior, the claw withdrawal time in the OA group showed a significant shorter time compared to the normal group in the cold‐plate paw withdrawal experiment (*p* < 0.05), while the claw lifting time in the OA+VA group was similar to the normal group.^[^ ^136^ ^]^ In a rabbit model of OA, an antifibrotic drug (pirfenidone) downregulated the transcriptome expression levels of COL1A1, TNF‐α and IL‐6 in synovial fibroblasts, leading to the suppression in synovial inflammation and fibrosis.^[^ ^137^ ^]^ In another in vitro experiment, the overexpression of EZH2 (Enhancer of Zest Homolog 2) aggravated the negative impact of IL‐1β on chondrocytes, and increased the expression of NO, PGE2, IL6, NGF and MMPs.^[^ ^138^ ^]^ Therefore, the inhibition of EZH2 reduced IL‐1β‐induced cartilage degradation. More impressively, intra‐articular injection of EZH2 inhibitors in arthritic mice inhibited cartilage degradation and increased joint motility in OA mice.^[^ ^138^ ^]^ Unlike inflammatory cytokines, MMPs secreted by synovial fibroblasts belong to a family of zinc‐dependent enzymes whose main function is to participate in cartilage matrix degradation.^[^ ^139^ ^]^ As a result, metalloproteinase inhibitors are considered as potential therapeutic targets, and previous studies have shown that these inhibitors can reduce the rate of articular cartilage degradation.^[^ ^140^, ^141^ ^]^ Bevacizumab, an anti‐VEGF drug, has been shown to reduce VEGF expression compared to controls (>2.5‐fold), which can inhibit angiogenesis and synovial hyperplasia, leading to reduced inflammation and cartilage degeneration in OA rabbits.^[^ ^142^ ^]^ In addition, the inhibition of some synovial cell‐associated signaling pathways may represent another target for the treatment of OA. It is well‐known that the Wnt/β‐catenin pathway is activated in synovium and cartilage in OA patients.^[^ ^143^ ^]^ Wnt proteins bind to their specific cell membrane receptors and participate in cell proliferation, differentiation, apoptosis and other biological processes through classical β‐linked protein‐dependent and non‐classical β‐linked protein‐independent signaling pathways, thus promoting the development of OA.^[^ ^143^ ^]^ A previous study showed that inhibition of the Wnt pathway using Wnt inhibitors (XAV‐939, C113) in a trauma model of OA showed a reduction in fibroblast proliferation and type I collagen synthesis in the ECM, thereby reducing arthritic symptoms.^[^ ^125^ ^]^ Similarly, zinc finger protein A20 reduces IL‐6 and IL‐8 release by inhibiting the NF‐κB pathway in synovial fibroblasts from OA patients.^[^ ^144^ ^]^ Recently, researchers found that enhancing the expression level of adenosine 3′,5′‐cyclic monophosphate in synovial fibroblasts attenuated TGF‐β‐mediated pro‐fibrotic responses and promoted the production of hyaluronic acid (HA) and proteoglycan‐4 (PRG4), demonstrating the involvement of HA and PRG4 in antifibrotic effects.^[^ ^145^ ^]^

Although the role of synovial cells in OA is gradually being recognized, and therapeutics targeting synovial cells are being investigated, most studies were limited to in vitro or in vivo in animal models. That being said, the inhibition of synovial inflammation, synovial congestion and angiogenesis using arterial embolization of the knee has been reported to be an effective and safe procedure for alleviating joint pain.^[^ ^146^, ^147^ ^]^ More importantly, many targeted therapeutic agents have been studied in relevant clinical trials (clinicaltrials.gov, Table 2).

**TABLE 2:** Clinical trials of targeted synovial cell therapy.

Target	Drug	Trial ID	Affected joint	Results	Phase	Ref.
NGF	Tanezumab (anti‐NGF antibody)	NCT00733902 NCT00744471 NCT00830063 NCT00863304 NCT00809354 NCT00864097 NCT00863772 NCT01089725 NCT00985621	Knee and hip	Effective improvement in pain and physical function, safe enough	3	[200]
	Tanezumab	NCT02709486	Knee and hip	Effective pain reduction, increased physical function	3	[201]
	Tanezumab	NCT01127893	Knee	Termination due to potential security issues	3	–
	Tanezumab	NCT02697773	Knee and hip	Significant improvement in joint pain and WOMAC score and physical function	3	[202]
NGF receptor tropomyosin‐related kinase A (TrkA)	GZ389988	NCT02424942 NCT02845271	Knee	Reduced pain with functional gain and an acceptable safety profile	2	[203]
IL	Anakinra (IL‐1 receptor antagonist)	NCT00110916	Knee	No significant improvement in OA symptoms	2	[204]
	AMG 108 (fully human monoclonal antibody to IL‐1R1)	NCT00110942	Knee	Mild clinical effects	2	[205]
	Lutikizumab (anti IL‐1α/β antibody)	NCT02384538	Hand	No improvement in pain or imaging outcomes	2	[206]
	Lutikizumab	NCT02087904	Knee	Poor improvement in WOMAC pain scores and synovitis	2	[201]
TNF‐α	Adalimumab	NCT00686439	Knee	May have therapeutic benefits for OA and needs to be further evaluated in controlled trials	1,2	[207]
	Adalimumab	NCT00296894	Hand	No improvement in joint pain, synovitis and bone marrow lesions	2	[208]
	Adalimumab	NCT00597623	Hand	Not better than a placebo in reducing pain	3	[209]
	Adalimumab	–	Knee	Effective and tolerated for moderate and severe knee OA	–	[210]
MMPs	PG‐530742 (MMPs inhibitor)	NCT00041756	Knee	Terminated by musculoskeletal toxicity	2	[203]
p38 MAP kinase	FX‐005 (p38 MAPK inhibitor)	NCT01291914	Knee	Better pain relief than control	1, 2	–
I‐kB kinase	SAR113945 (NF‐κB inhibitor)	NCT01113333 NCT01598415 NCT01511549 NCT01463488	Knee	No significant effect	1, 2	[211]
Wnt	Lorecivivint (Wnt inhibitor)	NCT03122860	Knee	Significant improvement in pain and function	2	[212]

#### 6.1.2 Targeted drug delivery systems

**FIGURE 6:**
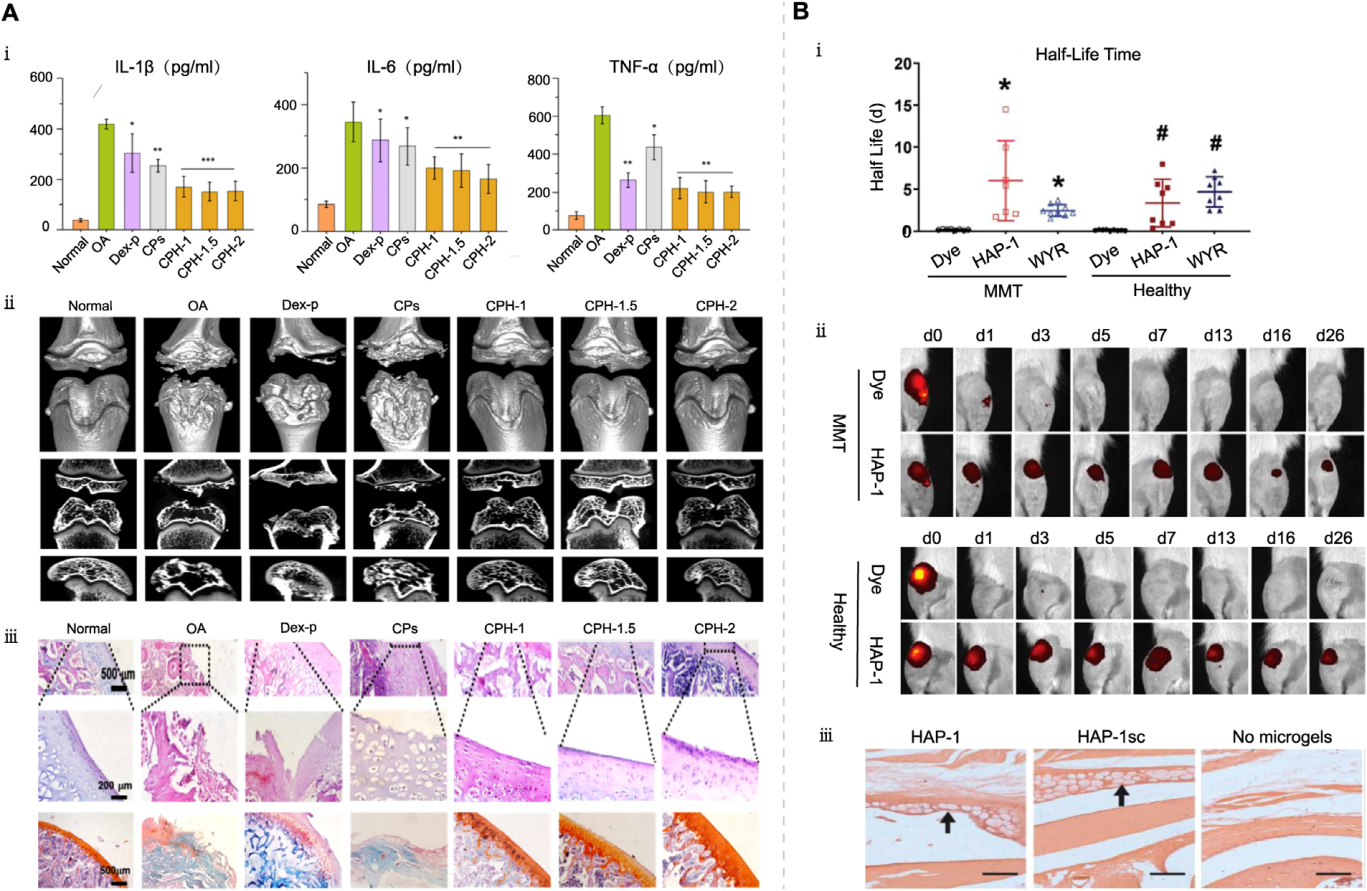
Drug delivery system targeting synovial cells for the treatment of OA. (A) The efficacy of multifunctional anti‐inflammatory drugs (CPHs) targeting synovial cells constructed by targeting ligand‐modified peptide dendrimer nanogels (PDN) for the treatment of OA. Reproduced with permission.^[^ ^148^ ^]^ Copyright 2021, Elsevier. (i) Expression levels of IL‐1β, IL‐6 and TNF‐α in joint treated with Dex‐p, CPs and CPHs. (ii) Efficacy of CPHs in treating OA, as shown by the micro‐CT 2D images of the rat knee joint, and by the quantitative measurements of micro‐CT bone remodeling. (iii) Efficacy of CPHs for OA, as shown by cartilage sections. Dex (dexamethasone), CPHs (targeted ligand modification), CPs (no targeted ligand modification). (B) Peptide‐functionalized nanocomposite PEG‐4MAL microgels prolong drug retention and precise targeting in the treatment of OA. Reproduced with permission.^[^ ^149^ ^]^ Copyright 2020, American Chemical Society. (i) Nanocomposite PEG‐4MAL microgels exhibit significantly higher retention times compared to free Cy7 dye. (ii) In vivo imaging systems of healthy and OA rat knees showed that free Cy7 dye was cleared from the joint faster than the nanocomposite microgels. (iii) Accumulation of peptide‐functionalized PEG‐4MAL nanocomposite microgels in synovium.

Recently, targeted drug delivery systems have gained popularity in the treatment of joint diseases (Figure 5). To target synovial macrophages and fibroblasts, Yang et al. developed peptide dendrimer nanogels (PDN) encapsulating CORM‐401. The surface of PDN was modified with folic acid (FA) and hyaluronic acid (HA) ligands for targeting purposes. Their result showed that PDN effectively targeted and bound to FA and HA receptors expressed by activated macrophages, rapidly releasing CO in response to high levels of intra‐articular H_2_O_2_ stimulation.^[^ ^148^ ^]^ CO inhibited macrophage proliferation, reduced the release of IL‐1β, IL‐6 and TNF‐α, can also rapidly depleted ROS in the OA joint cavity, thereby inhibiting cartilage degradation (Figure 6A).^[^ ^148^ ^]^ Similarly, the researchers designed nano‐composite 4‐arm‐poly (ethylene glycol)‐maleimide (PEG‐4MAL) microgels containing synovial cell‐binding peptides as well as poly (lactic acid–glycolic acid) nanoparticles (NPs).^[^ ^149^ ^]^ In a rat OA model, PEG‐4MAL microgels injected into the joint cavity remained in the joint for over 3 weeks and were precisely localized in the synovium (Figure 6B).^[^ ^149^ ^]^ In addition to synthetic nanoparticles, macrophage‐derived EVs also have been developed as nanocarriers for targeted delivery of immunosuppressive agents for the treatment of inflammatory arthritis.^[^ ^150^ ^]^

Targeted drug therapy, especially systemic drugs, can lead to severe toxic reactions, poor therapeutic efficiency and other side effects due to off‐target effects. Targeted drug delivery systems have the potential to address these drawbacks. Advanced nanocarriers such as liposomes, polymerics, dendrimers, exosomes, nanoparticles, nanocapsules and nanofibers, have been developed.^[^ ^151^, ^152^ ^]^ These nano‐sized carriers are commonly used in drug delivery systems owing to their high surface area‐to‐volume ratio for efficient drug loading, prolonged circulation time, good biocompatibility, degradability and targeting capacity. Nanocarriers can also be functionalized using hydrophilic polymers (such as polyethene glycol), ligands (such as proteins, peptides, aptamers) or cell membrane coating to construct biomimetic nanocarriers for improved targeting capability and reduced immunogenicity.^[^ ^153^, ^154^ ^]^ In addition, on‐demand released delivery systems designed by using internal (enzyme, ROS, PH) and external (temperature, light, ultrasound) stimulation factors can further improve the efficacy and reduce drug side effects.^[^ ^155^ ^]^ For instance, recently developed on‐demand release nanocarriers such as thermoresponsive polymer nanospheres,^[^ ^156^ ^]^ ROS‐sensitive nanoparticles,^[^ ^157^ ^]^ infrared light‐responsive chitosan (CS)‐modified molybdenum disulfide (MoS_2_) nanosheets^[^ ^158^ ^]^ have shown enhanced efficacy and reduced drug side effects in the treatment of OA as drug delivery systems. Therefore, surface functionalization using bionic ligands (e.g., folic acid, dextran sulfate and hyaluronic acid), and incorporation of biocompatible nanoparticles or nanocarriers targeting inflammatory cells or sites of inflammation could be the future direction.^[^ ^159^, ^160^ ^]^

### 6.2 SMSC‐based therapy for cartilage repair and inflammation inhibition

#### 6.2.1 SMSC therapy

Following their successful isolation, SMSCs have been applied to cartilage repair due to their stem cell properties. Different therapeutic strategies, including SMSC therapy (including direct SMSC transfusion or combination with tissue engineering approach) and SMSC‐derived EV therapy, have been developed (Figure 5).

The implanted autologous SMSCs were demonstrated in a femoral condyle of a micro mini pig model. Effective cartilage repair was observed in cartilage defects after treatment.^[^ ^161^ ^]^ Similarly, implantation of SMSCs in a rabbit model of cartilage defects significantly improved postoperative tissue quality, indicating the potential of SMSCs implantation to repair osteochondral damage.^[^ ^162^ ^]^ Another study, using an aged primate model, showed that autologous SMSCs contributed to the regeneration of the meniscus and slowed the progression of joint degeneration.^[^ ^163^ ^]^ Impressively, meniscus regeneration in a primate was also observed for the first time.^[^ ^163^ ^]^ In a recent clinical study of SMSCs for OA (UMIN 000026732), fully automated 3D MRI analysis of the joint before and after SMSCs injection in 14 patients showed a significant increase in cartilage thickness in the posterior medial region of the femoral cartilage after injection.^[^ ^164^ ^]^ In another clinical trial, Sekiya et al. implanted SMSCs into lesion defects in human knee cartilage through arthroscopy and also observed effective cartilage repair, MRI scores before and after treatment were 1.0 ± 0.3, 5.0 ± 0.7, and Lysholm scores before and after treatment were 76 ± 7, 95 ± 3.^[^ ^69^ ^]^

**FIGURE 7:**
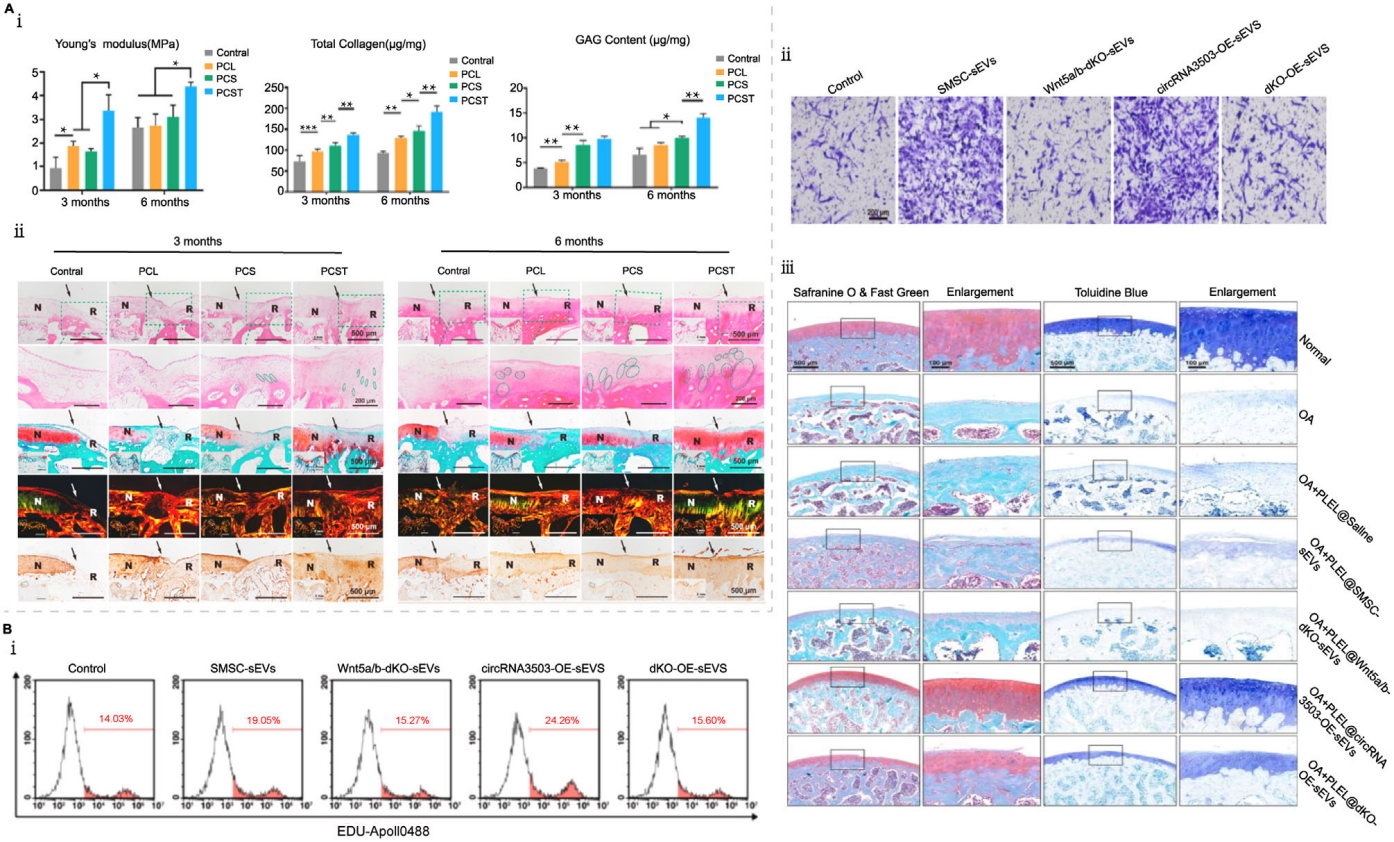
SMSC combines tissue engineering, nanotechnology, 3D printing and engineered EVs derived from SMSC for the treatment of cartilage defects. (A) Chitosan hydrogel/3D‐printed poly(ε‐caprolactone) (PCL) hybrid scaffold containing SMSCs for cartilage regeneration based on tetrahedral framework nucleic acid recruitment (TFNA). Reproduced with permission.^[^ ^169^ ^]^ Copyright 2021, Elsevier. (i) Efficacy of cartilage repair in the control and PCL groups, PCL/CS+SMSCs group (PCS) and PCL/CS+SMSCs+TFNA group (PCST), with better Young's modulus, GAG content and total collagen content in the PCST group. (ii) Histological (H&E, saffron‐O and Sirius red staining) and immunohistochemical analysis of the postoperative cartilage defect area showed the best results for PCLST repair. (B) SMSC‐derived EVs loaded with circRNA3503 combined with biogels to construct PLEL@circRNA3503‐OE‐sEVs nano‐delivery system for the treatment of OA. Reproduced with permission.^[^ ^187^ ^]^ Copyright 2021, Elsevier B.V. on behalf of KeAi. (i) The proliferative capacity of chondrocytes after treatment with different types of sEVs were measured by EdU assays. (ii) The migratory capacity of chondrocytes after treatment with different types of sEVs were measured by Transwell assays. (iii) In vivo experiments, histologic analysis with Safranin O & Fast Green and Toluidine Blue staining for different groups showed that PLEL@circRNA3503‐OE‐sEVs significantly inhibited the progression of OA. Wnt5a/b‐dKO‐sEVs: sEVs from SMSCs with Wnt5a and Wnt5b knockdown. dKO‐OE‐sEVs: sEVs from SMSCs overexpressing circRNA3503 with Wnt5a and Wnt5b knockdown.

With the development of biomedical engineering, the combined use of MSCs and scaffold implantations for cartilage repair has been introduced with enhanced therapeutic effect, due to the improved precision implantation and retention rate, together with the additional bioactivity and mechanical support provided by the scaffold. For example, rabbit synovial fluid derived MSCs encapsulated in injectable chitosan hydrogels were injected into cartilage defects in rabbit joints. Compared to the hydrogel scaffold group alone, the combined implantation of hydrogels with MSCs showed improved cartilage repair.^[^ ^165^ ^]^ In another study, a scaffold‐free tissue‐engineered construct (TEC) derived from SMSCs and a hydroxyapatite‐based artificial bone was used to implant cartilage defects in rabbit joints. Impressively, in the treatment group, cartilage tissue regained a similar mechanical property as native cartilage after 6 months.^[^ ^166^ ^]^ In a clinical trial, synovium was obtained arthroscopically and SMSCs were isolated and cultured into TEC matching the defect size. After the implantation, secondary arthroscopy and MRI confirmed that the defects had been successfully repaired. At 48 weeks postoperatively, the defect was completely covered in all patients, and no hypertrophy of the restored tissue was detected. The histological scores were (66–92, 80 ± 11, maximal value, 100).^[^ ^167^ ^]^ Arthroscopy, which is minimally invasive, will be an important technique for intra‐articular biomaterial implantation in the future. Koizumi et al. designed a scaffold‐free 3D TEC composed of SMSCs and cell‐synthesized ECM, demonstrating improved cartilage repair in human patients.^[^ ^168^ ^]^ Excitingly, this new technology is now in phase of clinical trials (UMIN000008266). Recently, combining tissue engineering and nanomaterials, a new cartilage regeneration system was developed. Chitosan hydrogel/3D‐printed poly (ε‐caprolactone) (PCL) hybrid scaffold was employed to encapsulate SMSCs and recruit tetrahedral framework nucleic acid (TFNA). The 3D‐printed PCL scaffold provided adequate mechanical support, and the TFNA provided a favorable microenvironment for enhanced proliferation and chondrogenic differentiation of the delivered SMSCs with improved cartilage repair (Figure 7A).^[^ ^169^ ^]^ In another study, electrospun nanofiber scaffolds combined with SMSCs‐derived tissue engineered construct (TEC) were used for meniscal repair in animal studies. The combined TEC nanofiber scaffolds significantly enhanced the repair of meniscal hoop structure and prevented cartilage degeneration compared to electrospun nanofiber scaffolds alone, demonstrating the feasibility of combined TEC nanofiber scaffolds as a potential tissue engineering approach to prevent cartilage degeneration.^[^ ^170^ ^]^

Biological scaffolds for tissue engineering must have good biocompatibility, mechanical stability to provide structural support while exhibiting controlled microstructure and adequate porosity, and biodegradability.^[^ ^171^ ^]^ Natural polymer scaffolds mainly include cell‐derived ECM, ECM components, and other natural molecules derived from non‐mammalian sources, such as chitosan, serine protein, alginate, and agarose.^[^ ^172^ ^]^ However, the disadvantages of natural polymers are evident. For example, cell‐derived ECM and hyaluronic acid (HA) affect cartilage repair due to their relatively poor mechanical properties.^[^ ^173^ ^]^ Synthetic polymers, on the other hand, have been greatly developed in the field of tissue engineering due to their excellent mechanical properties, and improved processing potential. Common synthetic polymers include poly (lactic‐co‐glycolic acid) (PLGA), poly (lactic acid) (PLA), poly (glycolic acid) (PGA) and poly (ethylene glycol) (PEG). However, synthetic materials often lack cell‐instructive biomolecules, requiring additives to improve their biofunctionality. Hence, an increasing number of research combines natural and synthetic polymers to enhance cellular response. For instance, scaffolds such as PLGA/articular cartilage‐derived ECM hybrid scaffolds,^[^ ^174^ ^]^ chitosan (CHT)/poly(ɛ‐caprolactone) (PCL) 3D hybrid scaffolds,^[^ ^175^ ^]^ have enhanced mechanical strength and cartilage repair properties. In recent years, advances in nanotechnology have led to the use of nanocomposites for biological tissue engineering with increased mechanical strength, biodegradability and biocompatibility, together with great modification potential, which is expected to be developed into more bionic nanoscaffolds.^[^ ^176^ ^]^ Both natural and synthetic polymeric materials can now be fabricated into nanofibers by different techniques, including electrospinning, self‐assembly and phase separation.^[^ ^177^ ^]^ For instance, electrospun nanoscaffolds composed of collagen or chondroitin sulfate and poly(ε‐caprolactone)‐poly(tetrahydrofuran) (PCL‐PTHF) have significantly enhanced stiffness, the chondrogenic potential of MSC compared to chondroitin sulfate scaffolds alone.^[^ ^178^ ^]^ Similarly, Sanchez et al. developed poly(lactic acid)/polyethylene glycol‐polyhedral oligomeric silsesquioxane (peg‐POSS/PLLA) nanocomposite scaffolds by electrostatic spinning. The addition of peg‐POSS produced a limited toxic reaction, significantly reduced the mean fiber diameter, increased the specific surface area of the scaffold, facilitated the adhesion and proliferation of MSCs, reduced the rate of scaffold degradation, and showed potential in cartilage tissue regeneration.^[^ ^179^ ^]^ In addition to their chondrogenic differentiation capability, SMSCs can reduce joint inflammation and promote cartilage repair through their immunomodulatory properties, elaborated in the above section. Taken together, SMSC implantation exhibits good cartilage and meniscal regenerative properties and is a reliable therapy for OA.

#### 6.2.2 SMSC‐derived EV therapy

Apart from the cells, EVs secreted by MSCs can also influence the biological functions of target cells. Previous research has shown that the overexpression of miR‐155‐5p exosomes by SMSCs could inhibit the progression of OA by promoting chondrocyte proliferation, reducing apoptosis, and regulating ECM secretion.^[^ ^180^ ^]^ In another example, Tao et al. demonstrated that SMSCs‐exosomes over‐expressing miR‐140‐5p enhanced chondrocyte proliferation and migration.^[^ ^181^ ^]^ Often lncRNAs, miRNAs or mRNAs within SMSCs exosomes will be transferred to target cells via endocytosis. miRNAs normally can bind 3′ UTR mRNAs to mediate the translational process. Meanwhile, lncRNAs bind to miRNAs competitively to affect the regulation of downstream gene expression by miRNAs.^[^ ^182^ ^]^ Compared to MSCs therapy, EVs derived from MSCs are considered to have better potential than MSCs therapy as cell‐free particles that are less affected by certain microenvironment or inflammatory environments, have more stable bioactivity and reduced immunogenicity.^[^ ^183^ ^]^ However, the rapid clearance effect of EVs, regardless of the systemically or locally delivery, is a challenge. Hence, researchers have introduced hydrogels to achieve control release purposes.^[^ ^184^ ^]^ Apart from using natural EVs to mediate cell‐to‐cell communication and promote cartilage repair, engineered EVs are also widely used as nano‐delivery systems.^[^ ^185^ ^]^ For instance, SMCS‐derived microvesicles loaded with tretinoin were applied to treat the rat OA model, showing enhanced anti‐inflammatory properties and promoting cartilage regeneration.^[^ ^186^ ^]^ Additionally, Tao et al. successfully isolated sEVs loaded with circRNA3503 (circRNA3503‐OE‐sEVs) from SMSCs, and combine with poly(d,l‐lactide)‐*b*‐poly(ethylene glycol)‐*b*‐poly (d,l‐lactide) (PDLLA‐PEG‐PDLLA, PLEL) triblock copolymer gels as novel therapeutics (PLEL@circRNA3503‐OE‐sEVs). Both in vitro and in vivo results revealed that circRNA3503‐OE‐sEVs overcame the side effects of Wnt5a/b in sEVs that inhibited cartilage ECM synthesis. The new systems also promoted cartilage ECM synthesis, inhibited degradation and reduced chondrocyte apoptosis (Figure 7B).^[^ ^187^ ^]^

To this end, SMSCs offer a potential alternative solution to treat joint disease and prevent the progression of OA. Particularly, with the advances in tissue engineering and nanotechnology, the combination of MSCs and MSC‐EVs with tissue engineering and nanomaterials is considered as a new trend in the development of future cartilage regeneration technologies, focusing on integrated treatments that improve biological stability, reduce drug side effects and enhance therapeutic efficacy.

## 7 FUTURE PERSPECTIVES AND CONCLUSIONS

The treatment of OA remains a significant challenge because of the avascular microenvironment of cartilage and the limited regenerative capacity of the chondrocytes. Although some hypothesis regarding the OA pathological development has been confirmed, the specific pathogenesis of the OA and the interaction on the cellular and molecular level during the onset and progression of OA remains unknown. Given the joint is a complex system and the importance of the synovium in maintaining cartilage function and joint homeostasis cannot be ignored, synovium and synovial cells have become a promising new trend for further research into the pathogenesis and treatment of OA. To date, the biological functions of the synovium and synovial cells, synovitis, synovial fibrosis and synovial angiogenesis, have been identified to be associated with OA inflammation, pain, swelling, and cartilage degradation.

Current clinical management together with drug and later‐stage surgical interventions, can only alleviate clinical symptoms and fail to regenerate the damaged cartilage tissue to slow or prevent the progression of OA. Given the important role of synovium or synovial cells in OA development, many studies target synovial fibroblasts and macrophages to regulate their paracrine factors for therapeutic purposes. In this respect, some progress has been made, including inhibition of synovial fibroblast and macrophage‐mediated inflammation, angiogenesis, fibrosis, pain and cartilage damage. These studies used drugs to inhibit the release of paracrine factors secreted by synovial fibroblasts and macrophages that cause inflammation, fibrosis, angiogenesis, pain, and cartilage damage. However, apart from anti‐NGF targeting drugs, other drugs have yet to be approved for clinical use due to their side effects, limited efficacy, and inadequate research investigation. Thus, improving the therapeutic effect of these targeted therapies could be a new research direction. Targeted drug delivery systems have tremendous advantages in terms of precise targeting capacity, reduced drug side effects, on‐demand release profile, and enhanced therapeutic effects, which could improve the therapeutic effect. For instance, folic acid‐modified polyethene glycol liposomes loaded with methotrexate (MTX) and catalase target activated macrophages to release drugs via catalase in response to ROS levels.^[^ ^188^ ^]^ Zhao et al. designed stearic acid‐octa‐arginine and folic acid decorated poly lactic‐*co*‐glycolic acid (PLGA)‐PK3‐based lipid polymeric hybrid pH‐responsive nanoparticles (Sta‐R8‐FA‐PPLPNs/MTX) targeting macrophages for inflammatory arthritis drug delivery therapy.^[^ ^189^ ^]^ In addition, exosome surface modification by folic acid–polyethene glycol (PEG)–cholesterol (Chol) compounds to construct exosome‐based bionanoparticles for drug delivery showed enhanced therapeutic efficacy and biocompatibility.^[^ ^190^ ^]^ Therefore, advanced drug delivery systems should be considered.

Furthermore, from a stem cell therapy point of view, SMSCs have been shown to possess good cartilage differentiation capacity along with the immunomodulatory capacity to mediate joint inflammation. In addition, SMSCs paracrine factors can inhibit apoptosis and promote chondrocyte proliferation and migration, contributing to the cartilage repair process. Consequently, SMSCs have been recognized as a promising approach to OA treatment. Compared to other popular stem cell sources in cartilage repair, namely BMSCs and AMSCs, limited evidence has shown that SMSCs would lose their chondrogenic potential due to aging or obesity, indicating the great clinical potential of SMSCs in OA treatment. That being said, the other advantages of SMSCs to replay other stem cells remain elusive, and the origin, together with its pathological conditions, may influence the chondrogenic potential of SMSCs. Therefore, further research is required. Using SMSCs together with advanced tissue engineering and nanotechnology has gained significant popularity. For example, SMSCs combined with 3D printed silk fibroin/gelatin (NP/ SF‐GEL) nanocomposite scaffold and Chitosan‐alginate composite 3D porous scaffold showed good cartilage repair capacity and biocompatibility.^[^ ^191^, ^192^ ^]^ Chitosan (CS)/polyvinyl alcohol (PVA)‐based nanofiber bionic scaffolds mimicked the extracellular matrix environment to promote the proliferation and differentiation of MSCs.^[^ ^193^ ^]^ That being said, improving the mechanical support, biocompatibility and degradability of the materials remains a clinical challenge due to the complex physiological microenvironment of human patients. Nanocomposite bionic scaffolds may be a trend for future research. As the technology advances, new 3D models and analysis techniques such as organoids and single‐cell sequencing allow researchers to better understand the pathological alteration during the OA development from a molecular and cellular level. Particularly, the subpopulations and differential expression of synovial cells at different stages of OA would be identified.

In conclusion, synovium and synovial cells have shown their contribution to joint function and the development of degenerative joint disease, namely OA. Although some synovial cell‐based therapies have been developed to treat OA, treatments targeting synovial fibroblasts or macrophages have shown unsatisfactory results in clinical trials. That being said, several SMSC‐based clinical trials have demonstrated promising therapeutic results, which could be the future focus. Since clinicians are gradually realizing the importance of synovium and synovial cells, new generation treatments based on synovial cells are expected in the near future.

## AUTHOR CONTRIBUTIONS

Zaijun Zou, Han Li, and Kai Yu contributed equally to this work. Kang Tian, Weiguo Zhang, and Xiaolin Cui selected the topic and guided the review. Zaijun Zou, Han Li, Kai Yu, and Xiaolin Cui wrote the manuscript. Zaijun Zou, Han Li, Kai Yu, Junnan Tang, Qiguang Wang, Guozhen Liu, Kang Tian, Weiguo Zhang, and Xiaolin Cui revised the manuscript. All authors read and approved the final manuscript.

## CONFLICT OF INTEREST STATEMENT

The authors declare no conflicts of interest.
